# Dysregulation of MMP-2 and MMP-9 in Post-COVID-19 and IPF: Correlations with Systemic Inflammation and Endothelial Dysfunction

**DOI:** 10.3390/jcm15020671

**Published:** 2026-01-14

**Authors:** Olga V. Balan, Irina E. Malysheva, Ella L. Tikhonovich, Liudmila A. Lysenko

**Affiliations:** 1 Centre for Biomedical Research, Karelian Research Centre of the Russian Academy of Sciences (KarRC RAS), 185910 Petrozavodsk, Russia; ovbalan14@gmail.com (O.V.B.); i.e.malysheva@yandex.ru (I.E.M.); 2 Respiratory Centre, Republic Hospital Named After V.A. Baranov, 185002 Petrozavodsk, Russia; tikhonovich.ella@mail.ru

**Keywords:** idiopathic pulmonary fibrosis, post-COVID-19 pulmonary fibrosis, long COVID, matrix metalloproteinase, MMP-2, MMP-9, endothelial dysfunction, endothelin-1, systemic inflammation, fibrosis

## Abstract

**Background/Objectives**: Post-COVID-19 pulmonary fibrosis (PCPF) and idiopathic pulmonary fibrosis (IPF) exhibit significant clinical and pathophysiological overlap, suggesting convergent molecular pathways driving fibrosis. This prospective longitudinal study investigates the sustained dysregulation of matrix metalloproteinases (MMP)-2 and MMP-9 and its relationship with evolving systemic inflammation and endothelial dysfunction in convalescent COVID-19 patients, with comparative analysis to IPF. **Methods**: We conducted a prospective observational study of 86 patients at 6 and 12 months post-SARS-CoV-2 infection, stratified by high-resolution CT evidence of PCPF (FB+ group, *n* = 32) or absence of fibrosis (FB− group, *n* = 54). Gene expression of *MMP-2* and *MMP-9* in peripheral blood leukocytes and circulating levels of MMP-2, MMP-9, pro-inflammatory cytokines (TNF-α, IL-6), and endothelial dysfunction markers (Endothelin-1 [ET-1], adhesion molecules) were quantified via qRT-PCR and ELISA. A pre-pandemic healthy control group (HD, *n* = 20) and an IPF patient group (*n* = 10) served as comparators. **Results**: A significant, sustained elevation of MMP-2 and MMP-9 was observed in all post-COVID-19 patients versus HDs, most pronounced in the FB+ group and qualitatively similar to IPF. A critical divergence emerged: FB− patients showed resolution of systemic inflammation (reduced TNF-α, IL-6), whereas FB+ patients exhibited persistent cytokine elevation. Critically, a delayed, severe endothelial dysfunction, characterized by a profound surge in ET-1 and elevated adhesion molecules, manifested exclusively in the FB+ cohort at 12 months. Positive correlations linked plasma MMP-2/9 levels with ET-1 (r_s_ = 0.65, *p* = 0.004; r_s_ = 0.49, *p* = 0.009) and ET-1 with sICAM-1 (r_s_ = 0.68, *p* = 0.01). **Conclusions**: The development of PCPF is associated with a distinct pathogenic triad: sustained MMP dysregulation, failure to resolve inflammation, and severe late-phase endothelial dysfunction. The correlative links between these components suggest a self-reinforcing loop. This systemic signature mirrors patterns in IPF, underscoring shared final pathways in fibrotic lung disease and identifying the MMP–inflammation–endothelial axis as a promising target for biomarker development and therapeutic intervention.

## 1. Introduction

The emergence of post-acute sequelae of SARS-CoV-2 infection (PASC), commonly termed “long COVID”, represents a formidable and lingering global health challenge stemming from the COVID-19 pandemic [1,2]. This syndrome encompasses a heterogeneous constellation of symptoms, including persistent fatigue, dyspnea, cognitive impairment, and cardiovascular complaints, that can endure for months to years following the initial acute infection, irrespective of its initial severity [3,4]. The pathophysiological foundations of long COVID remain enigmatic, with leading hypotheses implicating a complex interplay of potential mechanisms: reservoirs of persistent viral antigen or RNA triggering chronic immune stimulation [5,6,7,8]; widespread microvascular injury and endothelial dysfunction [9,10]; profound immune dysregulation and autoimmunity triggered by molecular mimicry [11,12]; and dysbiosis of the gut and other microbiomes [13]. Unraveling these mechanisms is not only critical for understanding long COVID but also provides a unique window into the fundamental biology of post-viral syndromes and chronic inflammatory disease.

Within the spectrum of long COVID, respiratory sequelae, and specifically the development of post-COVID-19 pulmonary fibrosis (PCPF), constitute a major source of long-term morbidity. Radiological studies indicate that a significant subset of patients hospitalized with severe COVID-19 develop persistent interstitial lung abnormalities, with a proportion progressing to a fibrotic lung disease phenotype characterized by traction bronchiectasis and honeycombing on high-resolution computed tomography (HRCT) [14,15]. Clinically, PCPF manifests with persistent restrictive lung physiology, impaired gas exchange, and reduced exercise tolerance. Intriguingly, PCPF shares striking clinical, radiological, and histopathological features with idiopathic pulmonary fibrosis (IPF), the prototypical and most lethal progressive fibrosing interstitial lung disease [16,17]. This overlap has prompted a vigorous scientific inquiry into whether these conditions converge on common pathogenic pathways, despite their distinct etiological origins: one being a virally triggered insult and the other arising from complex genetic–environmental interactions in a predisposed host.

Central to the processes of tissue injury, repair, and fibrosis are the matrix metalloproteinases (MMPs), a family of zinc-dependent endopeptidases responsible for the degradation and remodeling of the extracellular matrix (ECM) [18]. Their functions extend beyond ECM proteostasis to include the proteolytic activation or inactivation of cytokines, chemokines, growth factors, cell-surface receptors, and adhesion molecules [19,20]. This breadth of activity positions MMPs as master regulators of the tissue microenvironment, influencing inflammation, angiogenesis, cell proliferation, and apoptosis. The gelatinases MMP-2 and MMP-9 are of particular interest in lung and vascular biology. They are potent degraders of type IV collagen, a foundational component of basement membranes that underlies the alveolar epithelium and vascular endothelium [21]. Their deregulated activity is thus intimately linked to the loss of epithelial and endothelial barrier integrity, a hallmark of both acute lung injury and chronic fibrotic remodeling.

In the context of IPF, the role of MMPs is complex and context-dependent, often described as a “double-edged sword.” While certain MMPs (like MMP-7) are strongly upregulated and serve as prognostic biomarkers, the gelatinases are involved in both injurious tissue destruction and necessary repair [22,23]. Elevated levels of MMP-9, derived from alveolar macrophages and neutrophils, are consistently observed in bronchoalveolar lavage fluid and serum of IPF patients and correlate with disease progression [24]. MMP-2, often produced by fibroblasts and endothelial cells, contributes to the aberrant tissue remodeling and vascular changes associated with fibrosis [25]. In acute COVID-19, a profound dysregulation of the MMP system is a key feature of the pathogenic “cytokine storm” and tissue damage. Numerous studies report massively elevated plasma MMP-9 levels, which correlate strongly with disease severity, the need for mechanical ventilation, and mortality [26,27,28]. MMP-9, released primarily from activated neutrophils, contributes to endothelial injury, immunothrombosis, and the degradation of the alveolar–capillary barrier, fueling acute respiratory distress syndrome (ARDS) [29]. Interestingly, while MMP-2 is often decreased in acute COVID-19 plasma, possibly due to systemic consumption or dysregulated ACE/ACE2 balance, it is found to be upregulated in lung tissue, suggesting compartment-specific roles [30,31].

Beyond the lung, MMP-2 and MMP-9 are pivotal players in cardiovascular pathophysiology, including atherosclerosis, plaque rupture, and the development of arterial stiffness, a key marker of vascular aging and endothelial dysfunction [32,33]. A recent comprehensive review [34] meticulously details the distinct yet overlapping features of vascular diseases in COVID-19, highlighting the central role of endothelial injury and MMP-mediated vascular remodeling in both acute and post-acute phases [34]. This vascular pathology is not transient; emerging evidence indicates that endothelial dysfunction and oxidative stress persist for months after infection, particularly in women, suggesting a sustained insult to the vasculature that may underpin many long COVID symptoms [35,36].

The convergence of PCPF and IPF extends into molecular pathogenesis. Recent studies reveal significant immunological and proteomic overlaps, suggesting shared dysregulated immune and tissue remodeling pathways [37,38]. Proteomic analyses demonstrated an overlap in systemic proteins related to tissue remodeling and inflammation, solidifying the concept of common pathways [38]. Most compellingly, direct mechanistic evidence shows that key drivers of fibrosis, such as TGF-β1, phosphorylated Smad-2/3, Smad-7, and β-Catenin, are augmented in the pulmonary arteries of both IPF and, by inference, post-viral fibrotic conditions, actively promoting endothelial-to-mesenchymal transition (EndMT), a critical process in vascular remodeling and fibrosis [39]. This body of work strongly supports the hypothesis that PCPF and IPF, while initiated by different triggers, funnel into similar downstream profibrotic and pro-inflammatory cascades.

Despite the wealth of data on MMPs in acute COVID-19 and established IPF, critical gaps remain. The longitudinal trajectory of MMP dysregulation into the post-acute and chronic phases of COVID-19 is poorly mapped. It is unknown whether MMP elevation is a transient marker of acute injury or a sustained feature associated with specific long-term outcomes like PCPF. Furthermore, the relationship between persistent MMP activity, chronic systemic inflammation, and evolving endothelial dysfunction, a triad suggested by the shared pathophysiology of PCPF and IPF, has not been systematically investigated in a longitudinal cohort.

To address these gaps, we designed this prospective study with the following specific aims: (1) to longitudinally quantify the gene expression and plasma levels of MMP-2 and MMP-9 in patients at 6 and 12 months post-COVID-19, stratified by the presence or absence of radiological PCPF; (2) to characterize the concomitant longitudinal profiles of key pro-inflammatory cytokines (TNF-α, IL-6) and markers of endothelial dysfunction (ET-1, adhesion molecules); (3) to explore the correlations between MMP dysregulation, inflammation, and endothelial injury, testing the hypothesis of an interconnected pathogenic axis; and (4) to contextually compare these systemic profiles with those from a cohort of patients with established IPF, in order to identify shared molecular signatures of progressive fibrotic lung disease. We hypothesize that patients who develop PCPF will exhibit a distinct and persistent biochemical signature characterized by sustained MMP-2/9 elevation, failure to resolve systemic inflammation, and the emergence of endothelial dysfunction, forming a self-reinforcing loop that mirrors, in pattern not absolute magnitude, the dysregulation observed in IPF.

## 2. Materials and Methods

### 2.1. Ethics

This study was conducted in accordance with the ethical principles for medical research involving human subjects as outlined in the World Medical Association Declaration of Helsinki. The prospective study protocol, encompassing longitudinal sample collection from convalescent COVID-19 patients and laboratory analysis, was reviewed and approved by the Institutional Medical Ethics Committee of Petrozavodsk State University and the Ministry of Health of the Republic of Karelia (Protocol No. 2, dated 9 September 2024). The approval process for this comprehensive biomarker study was initiated following the initial clinical observation period. Prior to enrollment in the prospective biomarker component, written informed consent was obtained from each participant after a detailed explanation of the study’s aims, procedures, potential risks, and benefits.

### 2.2. Study Participants and Clinical Definitions

This prospective observational study employed a longitudinal, case–control design. The study population comprised three distinct groups:

1. Post-COVID-19 patients (total *n* = 86): adults with a prior laboratory-confirmed diagnosis of SARS-CoV-2 infection (via RT-PCR) during the years 2020–2021. The mean age of this cohort was 47.15 ± 0.84 years, with a 1:1 male-to-female ratio. All patients were recruited from the outpatient Respiratory Centre of the V.A. Baranov Republican Hospital in Petrozavodsk, Russia. Crucially, at the time of blood sampling for this study, all participants tested negative for active SARS-CoV-2 infection via RT-PCR.
2. Pre-pandemic Healthy Donors (HD, *n* = 20): this control group consisted of age-matched, conditionally healthy individuals with no known history of COVID-19 or chronic inflammatory diseases. Blood samples from this group were collected in 2019, prior to the onset of the SARS-CoV-2 pandemic, to establish a baseline unaffected by the virus. The mean age was 43.42 ± 1.14 years.
3. Idiopathic Pulmonary Fibrosis patients (IPF, *n* = 10): this comparator group consisted of patients with a multidisciplinary team-confirmed diagnosis of IPF based on current international guidelines, exhibiting a typical usual interstitial pneumonia pattern on HRCT and/or surgical lung biopsy. The mean age of this group was 63.6 ± 3.44 years. The significant age difference between the IPF and post-COVID-19 cohorts is acknowledged as a key confounder and is addressed in the Discussion.

Post-COVID-19 patients were stratified based on clinical and radiological assessments conducted at 3-6 months post-infection. Stratification was performed independently of the biomarker analysis. All patients underwent clinical evaluation and high-resolution computed tomography (HRCT). FB− Group—patients who had either no persistent lung abnormalities on HRCT or only showed transient, non-fibrotic sequelae (e.g., ground-glass opacities without traction bronchiectasis) that had resolved by the 12-month follow-up; these patients did not meet the clinical or radiological criteria for PCPF. FB+ Group (Post-COVID-19 Pulmonary Fibrosis—PCPF): patients with persistent HRCT findings consistent with fibrotic lung disease, according to established guidelines for post-COVID-19 lung abnormalities [40], including: (a) reticular opacities with peripheral and basal predominance; (b) traction bronchiectasis or bronchiolectasis within areas of opacity; (c) architectural distortion (e.g., fissural displacement, volume loss). To distinguish persistent fibrosis from slow-resolving organizing pneumonia, these findings were required to be present on at least two consecutive HRCT scans separated by a minimum of 3 months. Post-COVID-19 patients were then further categorized by the time of blood sampling relative to their initial infection, creating four subgroups for longitudinal analysis: FB− at 6 months (*n* = 30), FB+ at 6 months (*n* = 16), FB− at 12 months (*n* = 24), and FB+ at 12 months (*n* = 16).

To minimize confounding factors, stringent exclusion criteria were applied to all participants: concomitant active immunoinflammatory or chronic systemic diseases; acute infectious diseases (other than the COVID-19) within the preceding 6 months; current tobacco smoking or a history of significant tobacco use (>10 pack-years); alcohol abuse; body mass index (BMI) ≥ 28 kg/m^2^. Prior to biomarker analysis, all participants underwent routine hematological and biochemical screening (complete blood count, erythrocyte sedimentation rate, liver and kidney function tests, lipid profile; a BF-6800 hematological analyzer and a CS-300B automated biochemical analyzer, Dirui, Changchun, China) to ensure general metabolic health and cohort homogeneity. Values for all participants fell within or near the standard Biological Reference Intervals.

Detailed demographic and clinical characteristics of all study groups are presented in Table 1. This includes data on the severity of the initial COVID-19 illness (e.g., severity, hospitalization) and the presence of other common long COVID symptoms (fatigue, cognitive dysfunction).

### 2.3. Sample Collection and Processing

Peripheral venous blood samples were collected from all participants into vacuum tubes containing K3-EDTA as an anticoagulant. For post-COVID-19 patients, samples were drawn at the 6-month and 12-month follow-up visits. Plasma was obtained by centrifugation at 1500× *g* for 15 min at 4 °C within 2 h of collection, aliquoted, and stored at −80 °C until analysis. For RNA extraction, a separate whole blood aliquot was subjected to red blood cell lysis using a 0.86% ammonium chloride solution to isolate peripheral blood leukocytes (PBLs), which were then immediately processed for RNA extraction or stored in RNA stabilization reagent at −80 °C.

### 2.4. Enzyme-Linked Immunosorbent Assay (ELISA)

The plasma concentrations of the target proteins were determined using commercial, highly specific sandwich ELISA kits according to the manufacturers’ detailed protocols. All samples were assayed in duplicate, and the mean absorbance was used for calculation. MMP-2 and MMP-9: Quantified using Human MMP-2 and MMP-9 ELISA Kits (Sensitivity: <10 pg/mL; ELK Biotechnology, Wuhan, China). Endothelial markers, such as Endothelin-1 (ET-1), E-Selectin, soluble Intercellular Adhesion Molecule-1 (sICAM-1), and soluble Vascular Cell Adhesion Molecule-1 (sVCAM-1), were measured using corresponding Human ELISA Kits (ELK Biotechnology, Wuhan, China). Cytokine TNF-α and IL-6 levels were measured using TNFα-IFA-BEST and IL-6-IFA-BEST kits (Vector-Best, Novosibirsk, Russia). Plasma, rather than serum, was deliberately chosen for MMP and marker analysis to avoid the potential release of cellular contents (including MMPs from platelets and leukocytes) that occurs during the clotting process in serum preparation. Optical density was read on an AMR-100 microplate reader (Allsheng, Hangzhou, China).

### 2.5. RNA Extraction and Quantitative Real-Time PCR (qRT-PCR)

Total RNA was isolated from PBLs using MagZole universal reagent (Magen, Guangzhou, China) following the manufacturer’s instructions. RNA concentration and purity (A260/A280 ratio ~2.0) were assessed spectrophotometrically. To eliminate genomic DNA contamination, 1 μg of total RNA was treated with 1 unit of DNase I (Syntol, Moscow, Russia). Complementary DNA (cDNA) was synthesized from the DNase-treated RNA using an MMLV reverse transcriptase kit (Eurogen, Moscow, Russia) with random hexamer primers.

Quantitative real-time PCR was performed on a CFX96 Touch Real-Time PCR Detection System (Bio-Rad, Berkeley, CA, USA) using a SYBR Green-based master mix. Each reaction was run in a minimum of three technical replicates. The PCR protocol consisted of an initial denaturation at 95 °C for 3 min, followed by 40 cycles of 95 °C for 15 s and 60 °C for 30 s. A melt curve analysis was performed at the end of each run to confirm the specificity of amplification and the absence of primer–dimer artifacts. The primer sequences for the target genes (*MMP2*, *MMP9*) and the two reference genes (*18S rRNA*, *RPL19*) used for normalization are listed in Table 2. Primers were designed using Beacon Designer software (Premier Biosoft; https://www.premierbiosoft.com, accessed on 14 December 2024) and verified for specificity using NCBI BLAST tool (https://blast.ncbi.nlm.nih.gov, accessed on 25 December 2024).

The relative expression levels of *MMP2* and *MMP9* was calculated using the comparative ΔΔCt method [41]. The geometric mean of the Ct values from the two stable reference genes (*18S rRNA* and *RPL19*) was used for normalization, providing a robust internal control.

### 2.6. Statistical Analysis

Experimental data were processed using Microsoft Excel (version 2509, Microsoft Corporation, Redmond, WA, USA) and Statgraphics Centurion XVI software (version 16.2.04, Statgraphics Technologies, The Plains, VA, USA). According to the Shapiro–Wilk test, the distribution of the studied parameters was non-normal. Differences in gene expression levels and biochemical indices were evaluated using the Mann–Whitney U test. Correlation analysis was used to assess the relationships between parameters. Data are presented as median (Me) with 25th and 75th percentiles (Q1; Q3). The age of individuals included in the study is presented as mean ± standard error (M ± SE). Differences were considered statistically significant at *p* < 0.05.

## 3. Results

### 3.1. MMP-2 and MMP-9 Plasma Levels and Gene Expression

**Figure 1:**
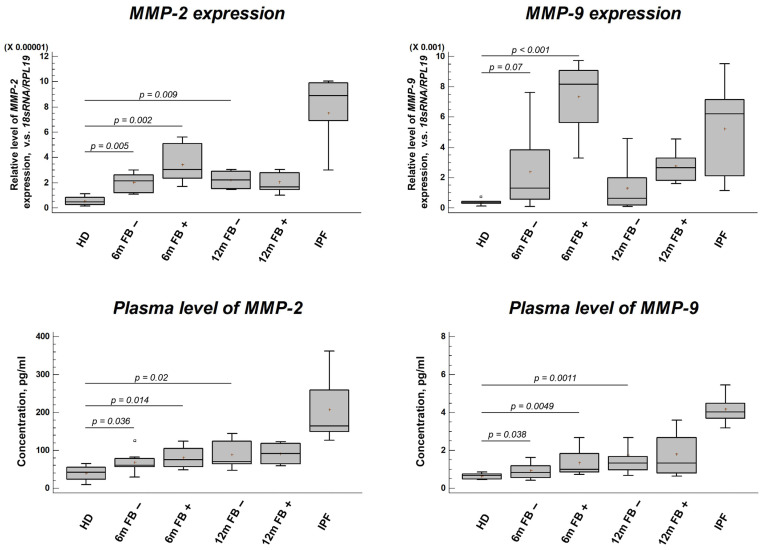
MMP-2 and MMP-9 Profiles in Post-COVID-19 and Idiopathic Pulmonary Fibrosis Patients. Gene expression of *MMP-2* and *MMP-9* in peripheral blood leukocytes (PBLs); circulating protein levels of MMP-2 and MMP-9 in plasma. Abbreviations: HD, healthy donors (pre-pandemic controls); 6m/12m, 6- or 12-months post-infection; FB−, patients without post-COVID-19 pulmonary fibrosis; FB+, patients with post-COVID-19 pulmonary fibrosis (PCPF); IPF, idiopathic pulmonary fibrosis. Data are presented as median with interquartile range; *p*-values are given in comparison with HD group.

Analysis of both gene expression in PBLs and corresponding protein levels in plasma revealed a significant and persistent dysregulation of the gelatinases MMP-2 and MMP-9 following COVID-19 (Figure 1).

At the transcriptional level, *MMP2* and *MMP9* mRNA expression was significantly elevated in PBLs from all post-COVID-19 patient groups (both FB− and FB+) at 6 and 12 months compared to pre-pandemic healthy donors (HDs) (*p* < 0.01 for all comparisons) (Figure 1). This increase was more pronounced in the FB+ groups, though the difference between FB+ and FB− at the same timepoint did not always reach statistical significance.

This transcriptional upregulation was faithfully reflected at the protein level. Plasma concentrations of both MMP-2 and MMP-9 were significantly higher in all post-COVID-19 groups compared to HDs (*p* < 0.05) (Figure 1). The FB+ groups consistently showed the highest median values. As expected, patients with established IPF demonstrated the highest levels of both MMPs, significantly exceeding those of the HDs (*p* < 0.001). While the median MMP levels in the FB+ groups were lower than in the IPF group, the pattern of co-elevation was qualitatively similar. Notably, there was no significant decline in MMP levels from the 6-month to the 12-month timepoint in either the FB− or FB+ cohorts, indicating a sustained rather than transient alteration.

### 3.2. Plasma Concentration of Pro-Inflammatory Cytokines

The plasma levels of the key pro-inflammatory cytokines, TNF-α and IL-6, demonstrated a clear divergence between patients with and without fibrotic sequelae (Table 3).

In patients without fibrotic sequelae (FB−), a significant reduction in cytokine levels was observed. At both 6 and 12 months post-infection, plasma concentrations of TNF-α and IL-6 in the FB− groups were significantly lower than those in the HD control group (e.g., TNF-α at 6m 0.64 pg/mL vs. HD 2.46 pg/mL, *p* = 0.0006), suggesting a resolution or even a subdued state of systemic inflammation compared to the pre-pandemic baseline. In contrast, patients diagnosed with PCPF (FB+) exhibited a significant and persistent elevation of these cytokines. TNF-α and IL-6 levels in the FB+ groups at both 6 and 12 months were significantly higher than in both the HD group and their FB− counterparts at the same timepoint (e.g., IL-6 in FB+ at 12m 4.66 pg/mL vs. HD 1.73 pg/mL, *p* = 0.0001). This indicates a failure to resolve the inflammatory response, resulting in a chronic, low-grade pro-inflammatory state specifically associated with the fibrotic outcome.

### 3.3. Plasma Concentration of Endothelial Dysfunction Markers

The analysis of markers reflecting endothelial cell activation and dysfunction uncovered a distinct and striking temporal pattern, predominantly in the FB+ group (Table 4). At the 6-month timepoint, most endothelial markers in the FB+ group were not significantly different from HD levels, with the notable exception of soluble ICAM-1 (sICAM-1), which was already elevated (1086.60 pg/mL vs. HD 724.54 pg/mL, *p* = 0.0006).

The most remarkable changes manifested at 12 months post-infection specifically in the FB+ cohort. This group exhibited a profound and significant increase in multiple markers. Plasma levels of Endothelin-1 (ET-1) surged to an exceptionally high median concentration of 404.13 pg/mL, nearly an order of magnitude greater than the HD group (50.82 pg/mL, *p* = 0.0042) and significantly higher than the FB− group at 12 months.

Soluble Adhesion Molecule sICAM-1 remained significantly elevated, and E-selectin also showed a significant increase (852.21 pg/mL vs. HD 604.08 pg/mL, *p* = 0.013). sVCAM-1 showed a trend towards increase in the FB+ group at 12 months but did not reach statistical significance compared to HDs.

In contrast, the FB− groups showed minimal changes in these endothelial markers at either timepoint, with only a mild, non-significant elevation in sICAM-1 at 12 months. This delineates a clear phenotype: the development of PCPF is associated with a delayed but severe systemic endothelial dysfunction, prominently featuring ET-1 dysregulation.

### 3.4. Correlations Between MMPs, Endothelial Dysfunction, and Inflammation

To explore potential interrelationships between the dysregulated pathways, we performed Spearman correlation analysis on the pooled biomarker data from post-COVID-19 patients. This revealed significant positive correlations of moderate strength between plasma MMP-2 levels and ET-1 levels (r_s_ = 0.65, *p* = 0.004), plasma MMP-9 levels and ET-1 levels (r_s_ = 0.49, *p* = 0.009), ET-1 levels and sICAM-1 levels (r_s_ = 0.68, *p* = 0.01).

These correlations suggest a linked axis where higher levels of proteolytic enzymes (MMPs) are associated with greater evidence of endothelial dysfunction (ET-1), which in turn is associated with increased endothelial activation (sICAM-1). No significant correlations were found between MMP levels and cytokine levels in this cohort.

## 4. Discussion

This longitudinal study provides a comprehensive analysis of the systemic molecular landscape during the post-acute phase of COVID-19, with a specific focus on differentiating patients who develop pulmonary fibrosis (PCPF) from those who recover without significant fibrotic sequelae. Our central findings delineate a distinct and clinically relevant pathogenic triad associated with PCPF: (1) sustained dysregulation of the gelatinases MMP-2 and MMP-9, (2) a failure to resolve systemic inflammation, characterized by persistent elevation of TNF-α and IL-6, and (3) the delayed emergence of severe endothelial dysfunction, dominated by an extreme surge in Endothelin-1 (ET-1). Furthermore, the significant correlations between MMPs, ET-1, and adhesion molecules suggest these pathways are not operating in isolation but are likely part of an interconnected, self-reinforcing biological loop that may drive ongoing injury and fibrotic remodeling. The qualitative similarity of this systemic signature to patterns observed in our IPF comparator group reinforces the concept of convergent final common pathways in progressive fibrotic lung disease, as increasingly highlighted in the recent literature [37,38,39].

Our findings align with and extend several key studies in the field. The observation of elevated MMP-9 in patients with persistent pulmonary pathology after COVID-19 was first reported by Lerum et al. [27], who linked it to high acute-phase viral load and weak antibody response. Our longitudinal design confirms that this MMP-9 dysregulation is sustained for up to 12 months. Importantly, we build upon this by simultaneously demonstrating a coordinated elevation of its counterpart, MMP-2, and by linking this proteolytic profile to distinct trajectories of systemic inflammation and endothelial dysfunction. Recent proteomic analyses, such as [38], have identified overlaps in systemic proteins related to tissue remodeling and inflammation between COVID-19 and fibrotic lung disease. Our study provides a focused, mechanistic complement to such broad profiling by demonstrating differential activation of a functionally linked axis (MMP-ET-1-inflammation) based on fibrotic outcome.

A key difference and strength of our study is its longitudinal, stratified design with two post-acute time points (6 and 12 months). In contrast, many biomarker studies in post-COVID-19 cohorts are cross-sectional or combine convalescent patients irrespective of specific organ sequelae [10,35]. By rigorously stratifying patients based on HRCT-defined PCPF and analyzing them at two time points, we were able to uncover the divergent evolution of systemic profiles. We show that biomarker levels in FB− patients trend toward normalization (for cytokines) or remain stably elevated (for MMPs), whereas in FB+ patients, they progress toward more severe dysfunction, particularly in the endothelial compartment. This temporal dimension is crucial for understanding the dynamics of post-COVID-19 pathology.

Furthermore, our cohort design inherently addresses the role of initial COVID-19 severity, as highlighted in Table 1. The FB+ (PCPF) group had a significantly higher proportion of patients who experienced moderate-to-severe acute infection requiring hospitalization (69%) compared to the FB− group (17%; *p* < 0.01). This aligns with the established link between severe initial pneumonitis and the risk of fibrotic sequelae [14,15] and suggests that the identified triad reflects an ongoing active pathological process in the post-acute phase, rather than merely being a legacy marker of past severe illness.

The sustained elevation of MMP-2 and MMP-9 we observed at both 6 and 12 months post-infection in all convalescent patients is a notable finding. It extends the well-documented acute-phase MMP dysregulation [26,27,28] into the chronic phase, challenging the notion that these proteolytic disturbances are transient. In patients without fibrosis (FB−), this persistent MMP elevation may represent a state of prolonged, low-grade tissue repair and immune remodeling. However, in the FB+ group, this dysregulation exists within a markedly different systemic context, suggesting a transition from adaptive repair to maladaptive remodeling. This distinction is crucial, as it clarifies that MMP elevation alone is not a specific biomarker for fibrosis, but becomes part of a pathogenic signature when coupled with other systemic disturbances. The pattern of MMP elevation in PCPF qualitatively resembled that in IPF, although absolute levels were lower. A key and unavoidable limitation in this comparison is the significant age difference between our IPF cohort (mean ~64 years) and our post-COVID-19 cohort (mean ~47 years). Age is a powerful confounder for MMP expression, inflammation, and endothelial function [42]. Therefore, while the IPF group serves as a useful clinical archetype for a progressive fibrotic phenotype, direct quantitative comparisons of absolute biomarker levels are not justified. The value of this comparison lies in the observed qualitative similarity, including the co-elevation of MMP-2, MMP-9, and inflammatory/endothelial markers, which underscores shared dysregulated pathways despite differing initiating events and demographics.

The divergent inflammatory profiles between FB− and FB+ patients provide critical insight. The significant reduction in TNF-α and IL-6 in FB− patients suggests a successful resolution of the acute hyperinflammatory state, a hallmark of recovery. Conversely, the persistent elevation of these cytokines in FB+ patients indicates a failure of inflammatory resolution, creating a chronic pro-fibrotic milieu. TNF-α is a potent inducer of epithelial cell apoptosis and fibroblast activation, while IL-6 drives Th17 cell differentiation and promotes fibroblast proliferation and collagen production [43,44]. This unresolved inflammation aligns with hypotheses of viral persistence [5,6,7,8] or infection-triggered immune dysregulation contributing to long COVID [11,12]. While our data do not directly prove an autoimmune etiology, they are consistent with a model of chronic immune activation that perpetuates tissue injury.

Perhaps the most striking finding of this study is the delayed, yet profound, endothelial dysfunction uniquely evident in the FB+ group at 12 months. The extreme elevation of ET-1, a potent vasoconstrictor, mitogen, and pro-fibrotic agent, is particularly noteworthy. ET-1 is known to stimulate collagen production in fibroblasts and is upregulated in the lungs of IPF patients, where it contributes to vascular remodeling and fibrosis [45,46]. The delayed onset suggests endothelial damage may be a cumulative process, or that dysfunctional endothelial cells become a sustained source of ET-1 during the fibrotic phase. This finding is strongly supported by recent work detailing persistent post-COVID-19 endothelial dysfunction and oxidative stress [35,36] and the overarching vascular pathology in COVID-19 sequelae [34]. The concomitant rise in E-selectin and sICAM-1 confirms widespread endothelial activation, facilitating leukocyte adhesion and transmigration, which can perpetuate local inflammation and injury.

The significant correlations we found between MMP-2/9 and ET-1, and between ET-1 and sICAM-1, suggest a plausible mechanistic link between these pathways, forming a potential feed-forward loop. MMPs, particularly MMP-2, are capable of processing the inactive ET-1 precursor into its active, potent form [47]. Conversely, ET-1 signaling can upregulate the expression of various MMPs, including MMP-2 and MMP-9, in endothelial and smooth muscle cells [48]. Furthermore, MMP-mediated cleavage of endothelial junctional proteins can increase vascular permeability, while shedding adhesion molecules like ICAM-1 can modulate leukocyte trafficking [19,49]. Thus, a vicious cycle can be envisioned: persistent inflammation and injury drive MMP activation; active MMPs process and activate ET-1 and disrupt endothelial integrity; active ET-1 further promotes vasoconstriction, fibrosis, and additional MMP production; and the damaged, activated endothelium releases more adhesion molecules, fueling more inflammation. This proposed MMP-ET-1-inflammation axis provides an integrative framework for understanding the systemic dysfunction in PCPF and aligns with the shared pathophysiology observed in IPF, where similar loops involving TGF-β, EndMT, and MMPs have been described [39].

Our study has several limitations. The sample size, especially for the FB+ and IPF groups, is modest, which may limit the generalizability of some findings and the power of subgroup analyses. The definition of PCPF was radiological; while we used strict HRCT criteria, the lack of comprehensive longitudinal pulmonary function tests or histopathological confirmation is a constraint. The measurement of circulating biomarkers, while informative of systemic status, may not fully reflect processes within the lung parenchyma. The observational design precludes definitive causal conclusions about the interactions within the proposed axis. Finally, as noted, the age discrepancy between the IPF and post-COVID-19 cohorts is a significant confounder that must be considered when interpreting comparative data.

These limitations point directly to essential future research directions. First, larger, multicenter prospective studies are needed to validate the prognostic value of this MMP–inflammation–endothelial signature for predicting fibrotic progression. Second, mechanistic studies in animal models of post-viral fibrosis or in vitro using lung endothelial cells and fibroblasts are required to experimentally dissect the causal relationships within the proposed pathogenic loop. Third, investigating therapeutic interventions that target this axis, such as endothelin receptor antagonists (already used in pulmonary arterial hypertension) or selective MMP modulators, could reveal novel strategies to prevent or mitigate PCPF. Our findings, by highlighting a targetable systemic loop, provide a strong rationale for such translational research.

## 5. Conclusions

In conclusion, this longitudinal study identifies a distinct systemic biochemical signature associated with post-COVID pulmonary fibrosis, characterized by the convergence of sustained MMP-2/9 dysregulation, unresolved chronic inflammation, and severe late-phase endothelial dysfunction, dominated by ET-1. The correlative links between these components suggest they form an interconnected pathogenic loop that may drive progressive fibrotic remodeling. While elevated MMPs are present in many convalescent patients, their occurrence within this broader triad of inflammation and endothelial injury appears to specifically delineate those at risk for poor fibrotic outcomes.

From a clinical perspective, these findings have several implications. First, the identified biomarkers, particularly the combination of MMP-2, MMP-9, ET-1, and persistent cytokines, could be developed into a plasma-based signature to help stratify patients at high risk for developing PCPF during follow-up, allowing for closer monitoring and earlier intervention. Second, the elucidation of the MMP-ET-1-inflammation axis provides a novel conceptual framework for understanding the pathophysiology of PCPF and its overlap with IPF. Most importantly, it reveals specific, therapeutically targetable pathways. Endothelin receptor antagonists (e.g., bosentan, macitentan) and strategies to modulate specific MMP activity or resolve chronic inflammation represent promising candidate approaches that warrant investigation in clinical trials for patients with progressive post-COVID-19 fibrotic lung disease. Thus, this work not only advances our understanding of long COVID sequelae but also translates mechanistic insight into tangible prospects for improved patient management and outcomes.

## Figures and Tables

**Table 1:** Baseline Demographic and Clinical Characteristics of Study Participants.

Characteristic	Healthy Donors (HD) (*n* = 20)	Post-COVID-19 FB− (*n* = 54)	Post-COVID-19 FB+ (PCPF) (*n* = 32)	IPF (*n* = 10)	*p*-Value
Age, years (mean ± SE)	43.42 ± 1.14	46.10 ± 1.1	48.8 ± 1.3	63.6 ± 3.44 *	<0.001 *
Sex, male/female (*n*)	10/10	26/28	16/16	6/4	0.85
BMI, kg/m^2^ (mean ± SE)	23.1 ± 0.5	24.3 ± 0.4	25.0 ± 0.6	25.8 ± 1.1	0.12
Smoking history, never/former	14/6	40/14	25/7	7/3	0.65
Initial COVID-19 severity:					<0.01
-Mild/ambulatory (*n*)	N/A	45	10	N/A	
-Moderate/severe, hospitalized (*n*)	N/A	9	22	N/A	
Time post-infection, months	N/A	6m: 30; 12m: 24	6m: 16; 12m: 16	N/A	N/A
Common long COVID symptoms:	0%	72%	88%	N/A	0.08
-Persistent fatigue	0%	65%	81%	N/A	
-Exertional dyspnea (mMRC ≥ 1)	0%	28%	94% *	100% *	<0.001
-Cognitive issues	0%	41%	56%	N/A	

Note: SE—Standard Error; BMI—Body Mass Index; mMRC—modified Medical Research Council dyspnea scale. N/A—not applicable. * Significant difference between IPF and other groups (HD, FB−, FB+), and also between the FB+ and FB− groups for the prevalence of exertional dyspnea.

**Table 2:** Primer Sequences used for qRT-PCR; F—forward, R—reverse.

Gene	Accession Number	Primer Sequences (5′ → 3′)	Amplicon Length (bp)
*MMP2*	NM_004530.6	F: ATTCTGGAGATACAATGAGGTGAAG R: GCACCCTTGAAGAAGTAGCTG	144
*MMP9*	NM_004994.3	F: CAGAGATGCGTGGAGAGTC R: AAGGCGTCGTCAATCACC	250
*18S rRNA*	NR_146119	F: AGAAACGGCTACCACATCCA R: CACCAGACTTGCCCTCCA	169
*RPL19*	NM_000981.4	F: AATCGCCAATGCCAACTC R: CCTTCCGCTTACCTATGC	155

**Table 3:** Plasma Levels of Proinflammatory Cytokines in Post-COVID-19 Patients Stratified by Fibrosis Status.

Protein	Healthy Donors	6 Months Post-COVID-19		12 Months Post-COVID-19	
	(*n* = 20)	FB− (*n* = 30)	FB+ (PCPF) (*n* = 16)	FB− (*n* = 24)	FB+ (PCPF) (*n* = 16)
TNFα, pg/mL	2.46 (1.89; 3.48)	**0.64** *** (0.52; 1.47)	**6.38** * (4.55; 7.27)	**0.91** *** (0.82; 1.18)	**5.86** ** (4.73; 7.28)
IL-6, pg/mL	1.73 (1.38; 2.17)	**1.00** * (0.63; 1.69)	2.25 (1.25; 2.50)	**1.13** *** (0.69; 1.13)	**4.66** *** (3.86; 5.45)

Data are Median (Q1; Q3). Bold indicates significant difference from HD control at * *p* < 0.05, ** *p* < 0.01, *** *p* < 0.001 (Mann–Whitney U test). FB− vs. FB+ comparisons at the same timepoint were also significant (*p* < 0.01) for both cytokines.

**Table 4:** Plasma Levels of Endothelial Dysfunction Markers in Post-COVID-19 Patients Stratified by Fibrosis Status.

Protein	Healthy Donors	6 Month Post-COVID-19		12 Month Post-COVID-19	
	(HD) (*n* = 20)	FB− (*n* = 30)	FB+ (PCPF) (*n* = 16)	FB− (*n* = 24)	FB+ (PCPF) (*n* = 16)
ET-1, pg/mL	50.82 (31.07; 99.37)	47.18 (37.00; 70.51)	50.54 (21.44; 81.20)	**124.16** * (98.47; 173.85)	**404.13** ** (392.47; 409.00)
E-selectin, pg/mL	604.08 (551.32; 758.87)	642.17 (544.21; 883.49)	686.25 (489.98; 884.93)	564.87 (530.62; 665.31)	**852.21** * (745.76; 958.66)
sICAM, pg/mL	724.54 (431.58; 808.15)	541.82 (462.92; 859.11)	**1086.60** *** (926.18; 1147.17)	**948.61** ** (852.12; 1237.01)	**1183.04** *** (1030.98; 1233.70)
sVCAM, pg/mL	0.20 (0.14; 0.50)	0.11 (0.09; 0.31)	0.12 (0.09; 0.39)	0.25 (0.09; 0.47)	1.56 (0.82; 2.29)

Data are Median (Q1; Q3). Bold indicates significant difference from HD control at * *p* < 0.05, ** *p* < 0.01, *** *p* < 0.001 (Mann–Whitney U test).

## Data Availability

All data generated or analyzed during this study are shown in this article.

## Abbreviations

The following abbreviations are used in this manuscript:

ARDS	acute respiratory distress syndrome
COVID-19	coronavirus disease 2019
ET-1	endothelin-1
HRCT	high-resolution computed tomography
IL	interleukin
IPF	idiopathic pulmonary fibrosis
MMP	matrix metalloproteinase
PBLs	peripheral blood leucocytes
PCPF	post-COVID-19 pulmonary fibrosis
SARS-CoV-2	severe acute respiratory syndrome coronavirus 2
sICAM	soluble intercellular cell adhesion molecule-1
sVCAM	soluble vascular cell adhesion molecule-1
TNFα	tumor necrosis factor-alpha
